# Altered functional connectivity of nucleus accumbens subregions associates with non‐motor symptoms in Parkinson's disease

**DOI:** 10.1111/cns.13979

**Published:** 2022-10-02

**Authors:** Lili Chen, Junling Wang, Mingrui Xia, Lianglong Sun, Junyan Sun, Linlin Gao, Dongling Zhang, Tao Wu

**Affiliations:** ^1^ Center for Movement Disorders, Department of Neurology, Beijing Tiantan Hospital Capital Medical University Beijing China; ^2^ State Key Laboratory of Cognitive Neuroscience and Learning Beijing Normal University Beijing China; ^3^ Beijing Key Laboratory of Brain Imaging and Connectomics Beijing Normal University Beijing China; ^4^ IDG/McGovern Institute for Brain Research Beijing Normal University Beijing China; ^5^ Department of General Medicine Tianjin Union Medical Center Tianjin China

**Keywords:** functional connectivity, nonmotor symptoms, nucleus accumbens, Parkinson's disease

## Abstract

**Aims:**

This study aimed to identify the functional connectivity (FC) changes of nucleus accumbens (NAc) subregions and characterize the association of network changes and non‐motor symptoms (NMS) in Parkinson's disease (PD).

**Methods:**

We enrolled 129 PD patients and 106 healthy controls from our center and the PPMI (Parkinson's Progression Markers Initiative) database. The FC of the bilateral core and shell of the NAc were measured and compared between the two groups. We further used partial least squares correlation to reveal the relationships between altered FC of NAc subregions and manifestations of NMS of PD.

**Results:**

The subregions of left core, left shell, and right core had reduced FC with extensive brain regions in PD patients compared with healthy controls. These three subregions were commonly associated with depression, anxiety, apathy, and cognitive impairment. Moreover, the left core and left shell were associated with excessive daytime sleepiness, whereas the right core was associated with olfactory impairment and rapid eye movement sleep behavior disorder.

**Conclusion:**

This study for the first time identified the neural network changes of NAc subregions in PD and the associations between network changes and phenotypes of NMS. Our findings provide new insights into the pathogenesis of NMS in PD.

## Graphical abstract

This original article explored the brain functional connectivity changes pattern of the nucleus accumbens subregions in Parkinson's disease for the first time and used the partial least squares correlation analysis to correlate the neural imaging changes with clinical symptoms, especially nonmotor symptoms. The relationship between the brain functional connectivity changes of each nucleus accumbens subregion and nonmotor symptoms of Parkinson's disease can provide new insights into the pathogenesis of nonmotor symptoms in Parkinson's disease.

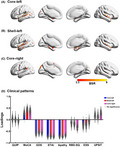

## 1 INTRODUCTION

Parkinson's disease (PD) is the second most common neurodegenerative disease, with more than 8.5 million individuals suffering from PD globally.^1^ PD is characterized by progressive dopaminergic neuron loss in the substantia nigra pars compacta and Lewy pathology. Nonmotor symptoms (NMS) are common in PD. Some NMS, such as hyposmia, rapid eye movement sleep behavior disorder (RBD), and constipation, begin long before cardinal motor symptoms and serve as prodromal biomarkers.^2^, ^3^ Cognitive and psychiatric problems can worsen with the progression and affect the quality of life of PD patients and caregivers.^2^ However, the neuromechanics of NMS in PD remains unclear, as a consequence, treatment of NMS is mainly symptomatic and the effect is not satisfactory.

The nucleus accumbens (NAc) is located in the ventral striatum. The NAc involves regulating appetitive or aversive behaviors and integrating cognitive and affective information processed by frontal and temporal areas.^4^ The core and shell are the main subregions of NAc and these two subregions play dissociable roles in approach and avoidance behaviors, cognition, and emotion.^5^ The core primarily promotes behaviors that effectively achieve motivation‐related goals, whereas the shell mainly inhibits behavioral patterns that may interfere with goal‐seeking.^4^ The core mainly receives the projections from the anterior cingulate gyrus (Broadman area [BA]24, BA32), anterior part of the basolateral amygdala, intralaminar and midline nuclei, and substantia nigra, whereas the shell mainly receives the projections from the ventromedial prefrontal cortex (PFC) (BA11), hippocampus (BA36), amygdala, thalamic subregions, and ventral tegmental area.^6^

Several functional MRI (fMRI) studies have investigated the role of NAc in disorders of the central neural system. EEG recordings provide high‐frequency functional analysis, whereas fMRI provides spatial resolution functional analysis in addition to structural connectivity,^7^ the temporal resolution of fMRI is lower than that of EEG. Reduced functional connectivity (FC) between the NAc and default mode network (DMN) has been detected in major depressive disorder patients,^8^ whereas increased FC between the NAc and DMN has been shown in bipolar disorder.^9^ The DMN is mainly composed of the medial prefrontal cortex, posterior cingulate cortex/precuneus, and lateral parietal cortex,^10^ which involves social behavior, mood control, and motivational drive. Hyperconnection between the ventral subiculum (BA36) and NAc involves in the development of schizophrenia, and disconnections between the ventral tegmental area, NAc, and hippocampus (BA36) were recorded in Alzheimer's disease.^11^

The role of NAc in the NMS of PD has received attention in recent years. Compared with healthy controls, the volume of NAc is significantly decreased in PD patients with mild cognitive impairment^12^ or with apathy.^13^ The reduced structural covariance between the left NAc and ipsilateral dorsolateral PFC (BA46) was associated with the severity of anxiety in PD patients.^14^ While these neuroimaging studies have suggested that structural and functional changes in the NAc are related to NMS in PD, the role of NAc subregions in NMS remains unclear. So far, only limited neuroimaging studies have focused on the association between NAc and NMS in PD, and whether the subregions of NAc have distinct roles in NMS of PD has not been investigated. Moreover, previous neuroimaging studies only focused on the relationship between NAc and a single NMS in PD.^12^, ^13^, ^14^ As multiple NMS always coexist in PD patients, modeling the relationship between NAc neural changes and multiple NMS manifestations in PD simultaneously makes it possible to clarify the relationship between NAc and different domains of NMS within one PD cohort.

Partial least squares (PLS) is a robust multivariate statistical technique that can analyze the correlation between brain structural/functional changes and clinical features.^15^ Rather than focusing on individual values of the data set, PLS correlation attempts to explain the relationship between brain structural/functional changes and clinical features. It aims to analyze the optimal linear combinations of these two data sets by deriving the latent variables. It has been widely used in neuroimaging studies, such as investigating the relationship between brain atrophy patterns and clinical features in de novo PD^16^ and the relationship between psychotic and misperception symptoms and imaging features in PD.^17^ Thus, this study aimed to clarify the association between functional network changes of NAc subregions and NMS manifestations using PLS correlation, to provide new insights into the pathogenesis of NMS in PD.

## 2 MATERIALS AND METHODS

### 2.1 Participants

This study included 129 PD patients and 106 age‐ and sex‐matched healthy controls (HC) (Supplementary Table S3). Of these subjects, 91 PD and 87 HC were from the Movement Disorders Center of the Tiantan Hospital of Capital Medical University, and 42 PD and 15 HC were from the PPMI (Parkinson's Progression Markers Initiative) database (www.ppmi‐info.org/data). The diagnosis of PD was according to the MDS Clinical Diagnostic Criteria.^18^ The exclusion criteria for HC included: a history of neurological or psychiatric disorders, a family history of movement disorders, and obvious cerebral lesions. The details of clinical features being assessed are provided in the Supplementary Materials and Methods. This study was conducted following the Declaration of Helsinki and was approved by the Institutional Review Board of Tiantan Hospital. All participants gave written informed consent before the study.

### 2.2 MRI data acquisition and preprocessing of imaging data

Our MRI data were acquired using a 3 T Magnetom Skyra scanner (Siemens, Erlangen, Germany) and PPMI data were collected on 3 T Siemens scanners. The scanning parameters of functional images and structure images can be found in the Supplementary Materials and Methods. Imaging data were preprocessed using a standard pipeline (Supplementary Materials and Methods).

### 2.3 Regions of Interest (ROIs)

We chose the bilateral core and shell of NAc as the ROIs (Figure S1). The core and shell were defined based on a probabilistic atlas of NAc subregions,^19^ which was created with 245 HC's MRI images from the Human Connectome Project.

### 2.4 Functional connectivity analysis

Because the data were obtained from different centers, ComBat was used to eliminate center/scanner effects. The bilateral core and shell of NAc were used as the seeds for FC analysis. We obtained reference time courses by calculating the average time course of each ROI. Correlation analysis was carried out by calculating the temporal correlation between the seed reference and the whole brain in a voxel‐wise manner. Then the individual correlation coefficient (*r*) maps were transformed into *z* maps using Fisher *r* to *z* transformation.

### 2.5 Statistical analyses

IBM SPSS Statistics 25 software was used for statistical analysis of demographic and clinical information. Kolmogorov–Smirnov‐test was used to check the distribution normality of all data. Chi‐square (*χ* ^2^) test was performed to evaluate the statistical significance of gender. The Mann–Whitney *U* test and two‐sample *t*‐tests were applied to measure the between‐group difference.

For FC analysis, two‐sample *T*‐tests were implemented to identify the FC differences between PD and HC in each ROI, with gender, age, education, and head motion as covariates. The significance of group differences was set at *p* < 0.05 with a false discovery rate (FDR) corrected. The extent threshold was 20 voxels. The results of the FC analysis were used for the subsequent PLS analysis.

### 2.6 Measurement of the ROI volumes

We used the Computational Anatomy Toolbox for SPM (CAT12, http://www.neuro.uni‐jena.de/cat/) for the structural images. The ANCOVA was used to measure the volume difference between PD and HC groups, with covariates of age, gender, and education (Supplementary Materials and Methods).

### 2.7 PLS analysis

**FIGURE 1:**
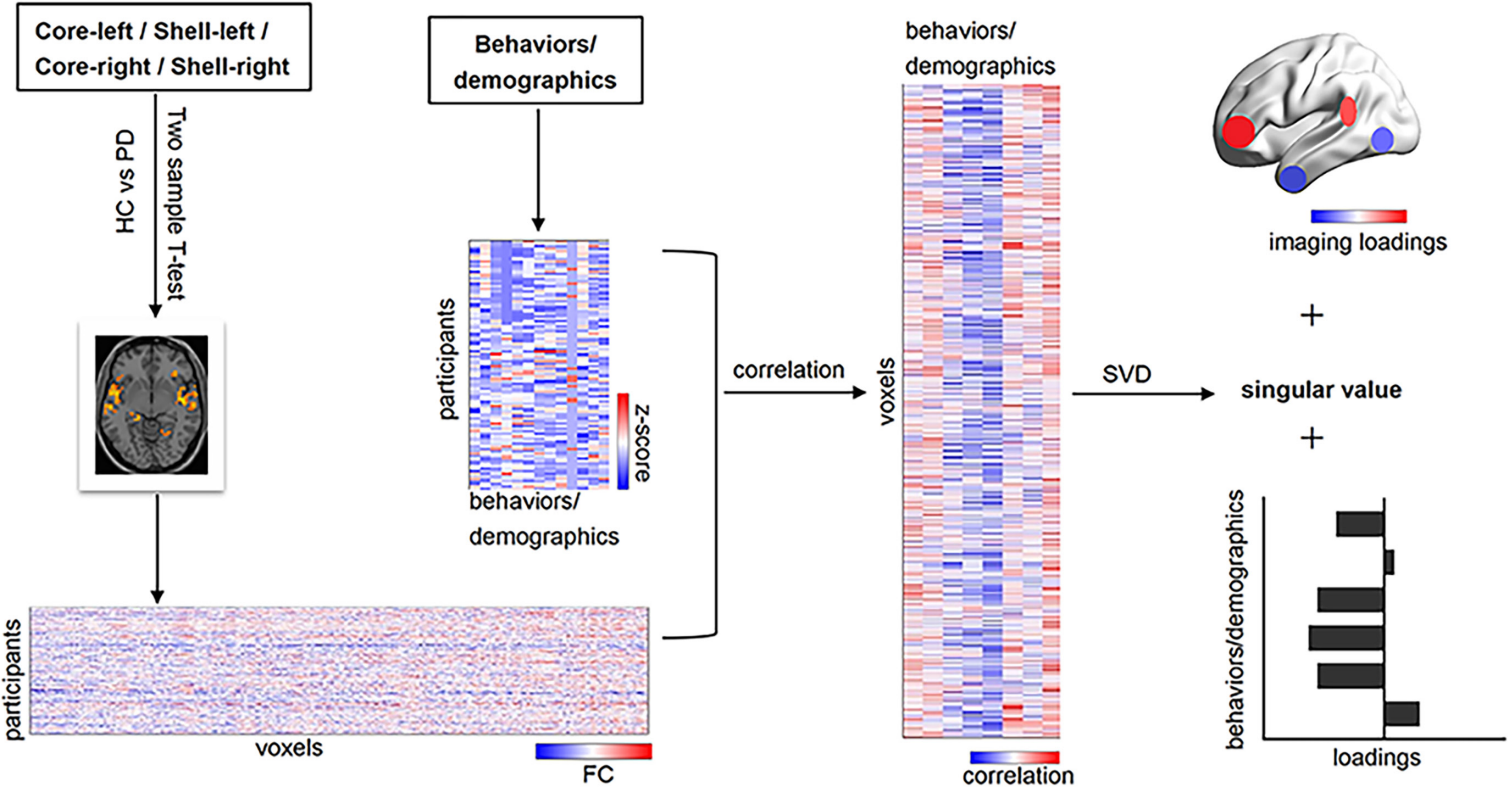
Partial least squares analysis flowchart. HC, healthy controls; PD, Parkinson disease group; FC, functional connectivity; SVD, singular value decomposition

PLS correlation was used to analyze the association patterns between the PD‐related FC network of each NAc subregion and the clinical features (Figure 1).^16^ The latent variable (LV) was derived from the singular value decomposition. The permutation test was used to evaluate the statistical significance (*N* = 1000 repetitions), and the significance was set as a *p* value of <0.05. Then, we selected the LVs with statistical significance for subsequent analysis.

Imaging and behavioral loadings were utilized to express the contribution of original voxels and behavioral features to the LVs, respectively. Bootstrap resampling was to test the significance of imaging and behavioral loadings with the replacement of 1000 times. The bootstrap ratio >3.3 of brain voxels was considered statistically significant (corresponding approximately to *p* < 0.001). The behavioral features were considered significant if the 95% confidence interval (CI) for its correlation coefficient did not cross zero. The details can be found in the Supplementary Materials and Methods.

## 3 RESULTS

### 3.1 Demographic and clinical information

Thirteen PD patients and 6 HCs were excluded due to excessive head motions, so this study finally included 116 PD patients and 100 HCs. All demographic and clinical information of the remaining participants is shown in Table 1. No significant differences in gender, age, education, TIV, gray matter volume, and volume of NAc subregions were noted between PD patients and HCs. The GDS, STAI, UPDRS‐I‐Apathy, UPSIT, MoCA, RBD‐SQ, and ESS scores were significantly different between the groups.

**TABLE 1:** Demographic and clinical features of participants

	PD (mean ± SD)	HC (mean ± SD)	*p* value
Age (years)	59.51 ± 9.40	60.40 ± 8.56	0.470
Gender (male/female)	59/57	45/55	0.390^a^
Duration (years)	3.68 ± 3.74	NA	
LEDD (mg)	312.46 ± 306.92	NA	
UPDRS‐III	25.66 ± 12.12	NA	
H&Y stage	1.72 ± 0.59	NA	
QUIP	0.1034 ± 0.30586	0	
MoCA	24.41 ± 4.15	26.03 ± 2.75	0.007
GDS	2.76 ± 2.38	0	0.000
STAI	36.03 ± 10.40	27.77 ± 3.99	0.000
UPDRS‐I‐Apathy	0.5603 ± 0.79	0.02 ± 0.14	0.000
RBD‐SQ	4.74 ± 2.69	3.76 ± 1.90	0.024
Epworth	5.56 ± 4.56	3.90 ± 2.34	0.041
UPSIT	24.74 ± 9.20	34.43 ± 3.46	0.000
Core‐left (mm^3^)	376.90 ± 47.22	378.71 ± 39.29	0.467
Shell‐left (mm^3^)	503.36 ± 62.30	503.31 ± 46.38	0.646
Core‐right (mm^3^)	296.17 ± 37.05	298.91 ± 34.60	0.355
Shell‐right (mm^3^)	598.93 ± 67.59	600.88 ± 57.39	0.449
TIV (cm^3^)	1485.50 ± 137.33	1449.29 ± 132.86	0.085
GM (cm^3^)	641.89 ± 57.13	644.44 ± 51.38	0.265

Abbreviations: Core‐ right, the volume of right core in nucleus accumbens; Core‐left, the volume of left core in nucleus accumbens; GDS, Geriatric Depression Scale‐15; GM, gray matter volume; LEDD, Levodopa equivalent daily dose; MoCA, Montreal Cognitive Assessment; QUIP, Questionnaire for Impulsive‐Compulsive Disorders in Parkinson's Disease; RBD‐SQ, REM Sleep Behavior Disorder Screening Questionnaire; Shell‐left, the volume of left shell in nucleus accumbens; Shell‐right, the volume of right shell in nucleus accumbens; STAI, State–Trait Anxiety Inventory, T‐AI part; TIV, the total intracranial volume; UPDRS‐I‐Apathy, Movement Disorder Society Unified Parkinson's Disease Rating Scale, part I, Question for apathy; UPDRS‐III, Movement Disorder Society Unified Parkinson's Disease Rating Scale, part III; UPSIT, University of Pennsylvania Smell Identification Test.

^a^ Chi‐square test.

### 3.2 Functional connectivity analysis

**FIGURE 2:**
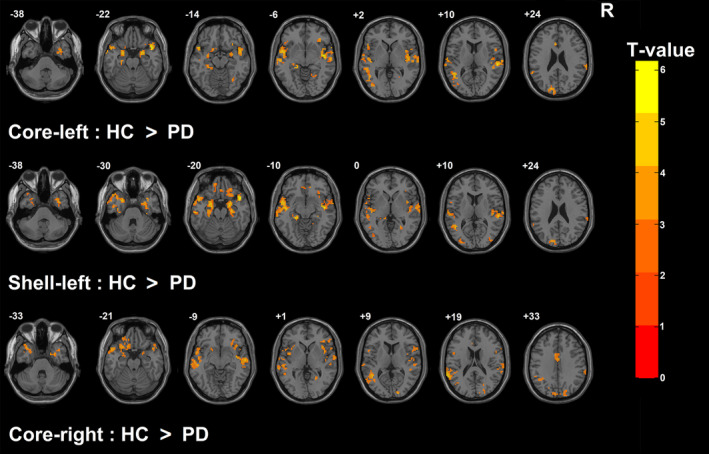
Results of functional connectivity. Brain regions with reduced functional connectivity with the left core, left shell, and right core of NAc in PD patients compared with HC. The threshold for display was set to *p* < 0.05 with a false discovery rate (FDR) corrected, and the extent threshold was 20 voxels. For further details, see Supplementary Table S1. HC, healthy controls; PD, Parkinson's disease; NAc, nucleus accumbens

The differences in FC between PD and HC groups in each NAc subregion are shown in Figure 2. The left core, left shell, and right core commonly had reduced FC with the bilateral temporal lobe, right insula, right parahippocampal gyrus, right fusiform gyrus, right lingual gyrus, left inferior frontal gyrus, left superior occipital gyrus, and left cuneus in PD patients compared with HC.

In addition, the left core showed decreased FC with the right precentral gyrus in PD patients compared with HC. The left shell had reduced FC with the left fusiform gyrus, right middle occipital gyrus, right OFC, right rectus gyrus, right ventromedial PFC, and right hippocampus in PD patients compared with HC. The right core showed decreased FC with the left supramarginal gyrus, left angular gyrus, left supplementary motor area (SMA), left OFC, left superior and middle frontal gyrus, left superior and inferior parietal gyrus, left precentral gyrus, left insula, right middle temporal gyrus, right superior occipital gyrus, right cuneus, and right inferior frontal gyrus in PD patients compared with HC (Supplementary Table S1). We did not find significant between‐group differences in FC in the right shell (*p* > 0.05, FDR corrected). Thus, PLS analysis was only conducted in the left core, left shell, and right core.

### 3.3 PLS analysis

#### 3.3.1 Clinical features relating to FC changes

For each subregion, we identified one statistically significant latent variable (LV‐I) related to clinical information and their corresponding PD‐related FC network (left core: 63% of total covariance, *p* < 0.001; left shell: 62% of total covariance, *p* < 0.001; right core: 48% of total covariance, *p* < 0.005). These LV‐Is were selected for further analysis (Figure S2).

For all subregions, the contributors to LV‐I included gender, education, disease duration, LEDD, UPDRS‐III, H&Y, TIV, gray matter volume, the volume of the subregion, the cognitive status (MoCA), anxiety (STAI), apathy (UPDRS‐I), depression (GDS), RBD (RBD‐SQ), and olfactory impairment (UPSIT) (Figures S3–S5), and age was also a contributor to LV‐I in the left core (Figure S3a).

#### 3.3.2 NMS phenotypes relating to FC changes

**FIGURE 3:**
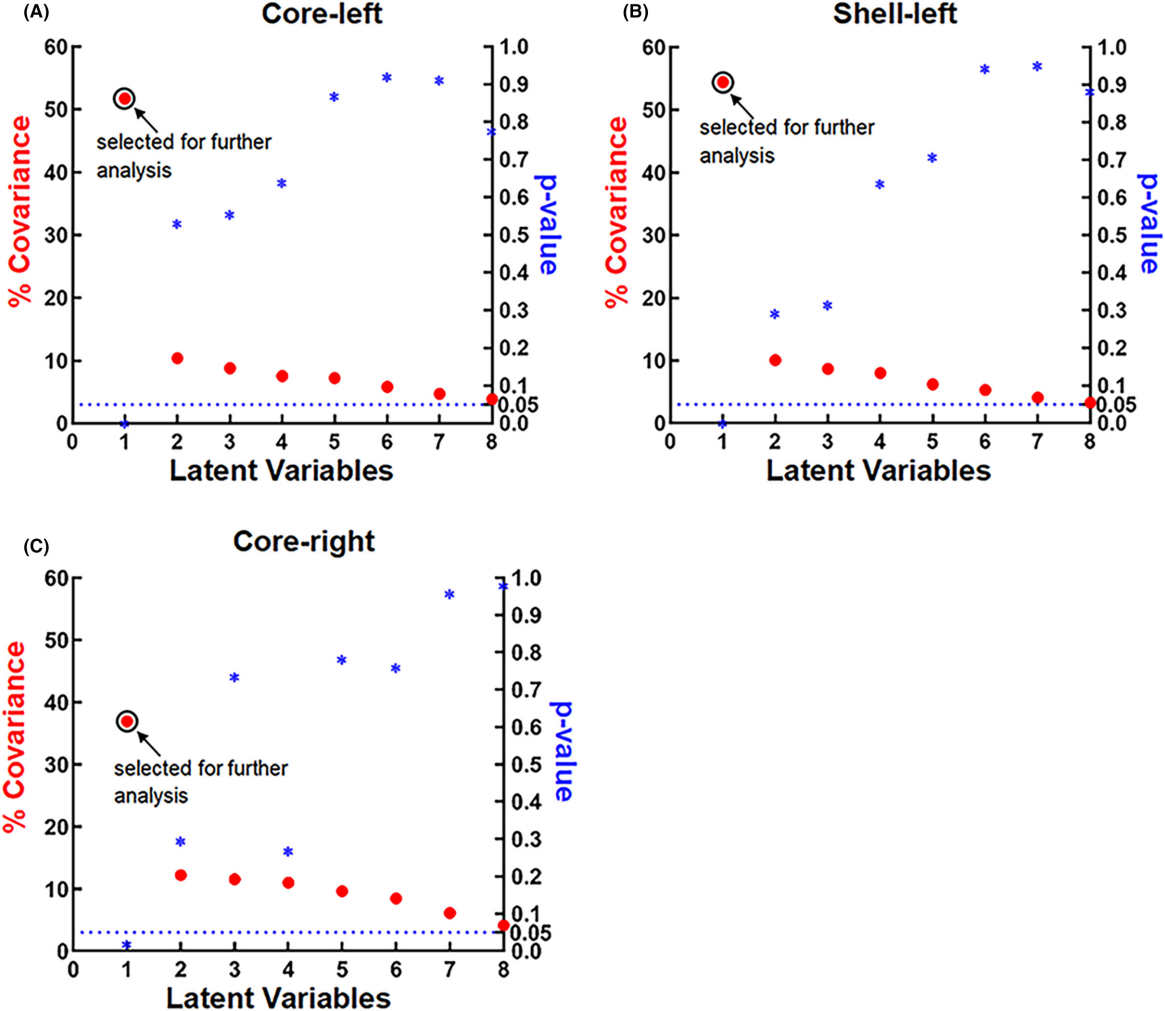
Covariance explained and permutation *p*‐values. Covariance explained and permutation *p*‐values for all latent variables in the PLS analysis after removing age, education, disease duration, LEDD, UPDRS‐III, H&Y, the volume of subregions, TIV, and gray matter volume in the left core (A), left shell (B) and right core (C). The first latent variable (LV‐I) is selected for further analysis.

We repeated the PLS analysis after removing the effects of age, education, disease duration, LEDD, UPDRS‐III, H&Y, the volume of subregions, TIV, and gray matter volume to ensure the specificity of contributions of FC changes of NAc subregions to the NMS.^16^ As a result, only one significant LV was found in each subregion (left core: 52% of total covariance, *p* < 0.001; left shell: 54% of total covariance, *p* < 0.001; right core: 37% of total covariance, *p* < 0.05) (Figure 3).

**FIGURE 4:**
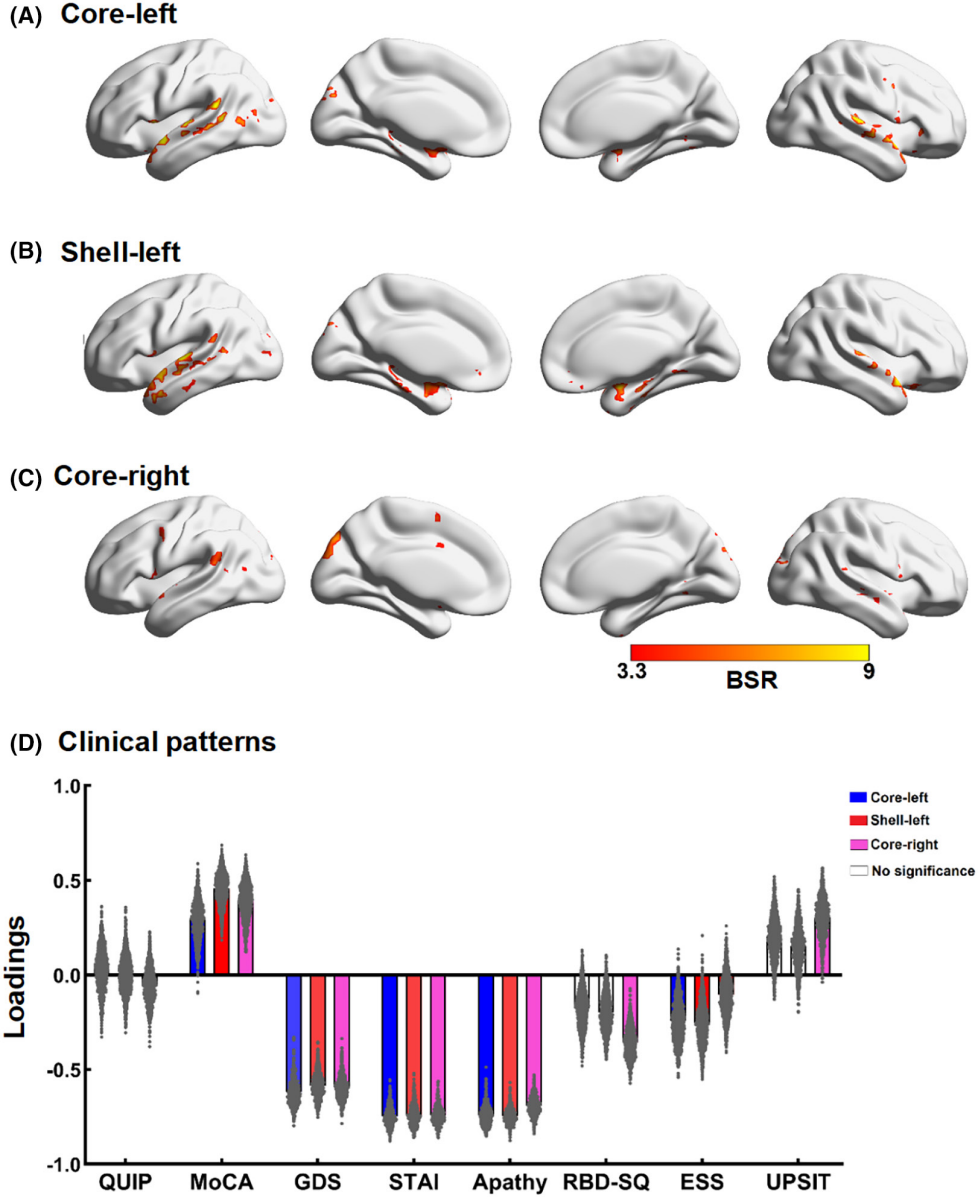
The FC‐behavior pattern in PLS results. The FC‐behavior pattern of left core, left shell, and right core with covariates. Brain imaging pattern bootstrap ratios in MNI space of left core (A), left shell (B), and right core (C). And more imaging pattern details see Supplementary Table S2. (D) Clinical behavioral pattern. Significant variables associated with the pattern are indicated in colors. BSR, bootstrap ratios; QUIP, Questionnaire for Impulsive‐Compulsive Disorders in Parkinson's Disease; MoCA, Montreal Cognitive Assessment; GDS, Geriatric Depression Scale‐15; STAI, State–Trait Anxiety Inventory, T‐AI part; Apathy, Movement Disorder Society Unified Parkinson's Disease Rating Scale, part I, Question for apathy; RBD‐SQ, REM Sleep Behavior Disorder Screening Questionnaire; ESS, Epworth Sleepiness Scale; UPSIT, University of Pennsylvania Smell Identification Test

For the left core, the strongest contributor to LV‐I was anxiety (*R* = −0.75, 95% CI [−0.83, −0.63]), followed by apathy (*R* = −0.74, 95% CI [−0.83, −0.63]). Depression (*R* = −0.62, 95% CI [−0.74, −0.47]), cognitive impairment (*R* = 0.29, 95% CI [0.09, 0.48]), and excessive daytime sleepiness measured by ESS (*R* = −0.24, 95% CI [−0.45, −0.03]) were other significant contributors to LV‐I (Figure 4D). As for the left shell, the strongest contributor to LV‐I was apathy (*R* = −0.74, 95% CI [−0.82, −0.65]), followed by anxiety (*R* = −0.74, 95% CI [−0.83, −0.61]) and depression (*R* = −0.58, 95% CI [−0.70, −0.45]). Cognitive impairment (*R* = 0.46, 95% CI [0.28, 0.61]) and excessive daytime sleepiness (*R* = −0.25, 95% CI [−0.46, −0.04]) were other significant contributors to LV‐I (Figure 4D). For the right core, the strongest contributor to LV‐I was anxiety (*R* = −0.74, 95% CI [−0.82, −0.63]), followed by apathy (*R* = −0.69, 95% CI [−0.78, −0.58]), and depression (*R* = −0.59, 95% CI [−0.71, −0.46]). Cognitive impairment (*R* = 0.39, 95% CI [0.20, 0.56]), RBD (*R* = −0.36, 95% CI [−0.50, −0.19]), and olfactory impairment (*R* = 0.30, 95% CI [0.10, 0.49]) were other significant contributors to LV‐I (Figure 4D).

#### 3.3.3 FC patterns associated with NMS

The pattern of FC changes corresponding to the clinical features in LV‐I in each subregion is shown in Figure 4A–C and Supplementary Table S2. The reduced FC between the three subregions of NAc and the bilateral superior temporal gyrus, bilateral superior temporal pole, left middle temporal gyrus, left superior occipital gyrus, left cuneus, right lingual gyrus, left inferior frontal gyrus‐opercular part, and right insula was associated with the NMS manifestations in PD. Moreover, each subregion had unique network changes in the imaging–behavior correlation pattern. The reduced FC between the left core and right precentral gyrus, right inferior frontal gyrus‐pars orbitalis, and right middle frontal gyrus was associated with NMS features. The decreased FC between the left shell and right hippocampus, left inferior temporal gyrus, right post‐OFC, bilateral ventromedial PFC, and the bilateral middle temporal pole were associated with LV‐I in PD. In addition, the reduced FC between the right shell and the right middle temporal gyrus, right superior occipital gyrus, left superior frontal gyrus, left superior parietal gyrus, left inferior parietal gyrus, left middle cingulate cortex, right cuneus, left precentral gyrus, right calcarine fissure, left supramarginal gyrus, and left SMA was associated with the NMS features in PD (Supplementary Table S2).

**FIGURE 5:**
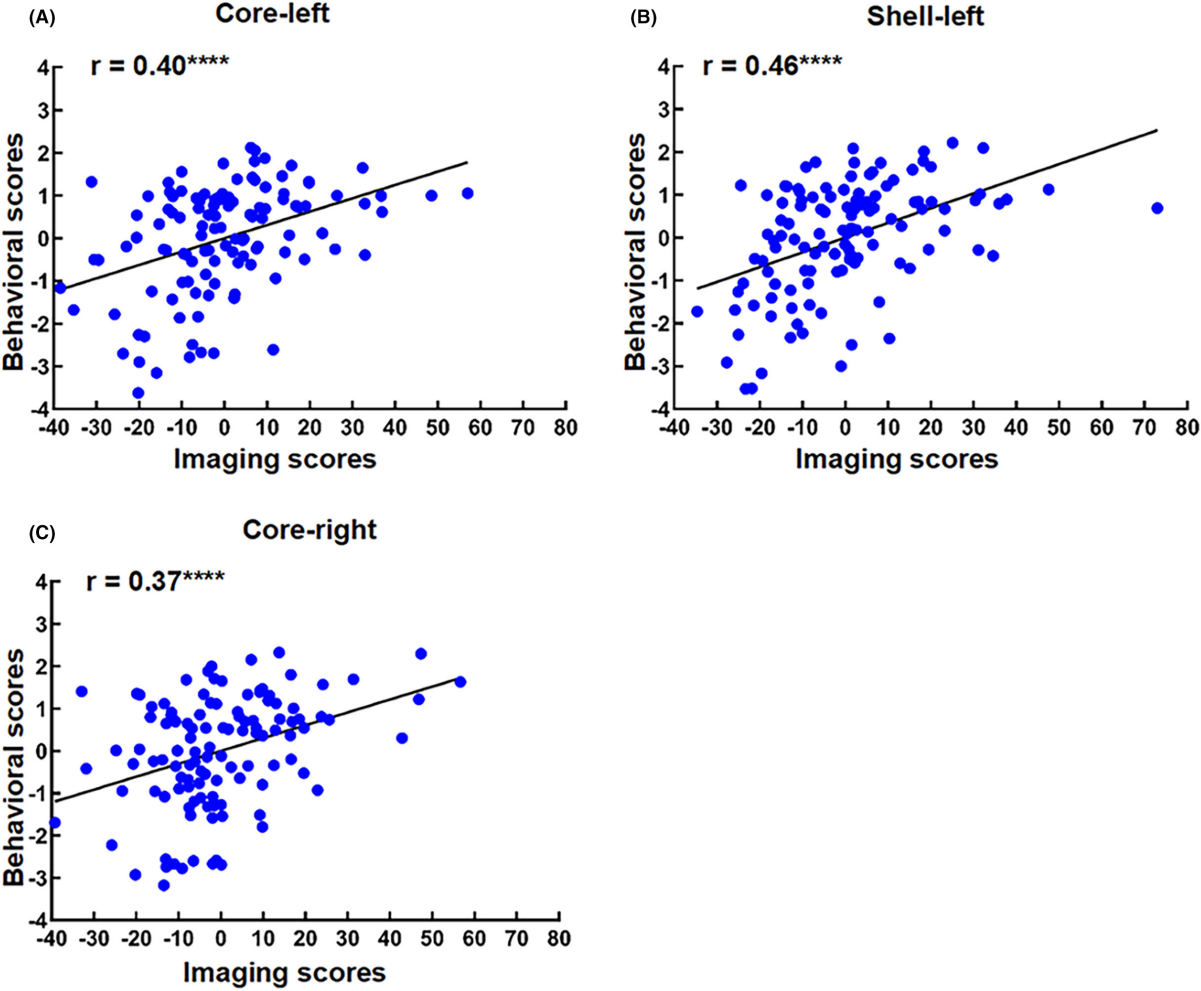
Correlation between imaging and behavioral scores. Correlation between imaging and behavioral scores in the left core (A), left shell (B), and right core (C). Pearson correlation coefficient (*r*) indicates correlation. ****p < 0.0001

The imaging and behavioral scores were correlated (left core: *r* = 0.40; left shell: *r* = 0.46; right core: *r* = 0.37) (Figure 5), which reflect the weighted pattern of individual patient's contributions to the imaging and behavioral scores and indicate that PD patients with greater reduction of network connectivity in NAc are more likely to have these NMS.

## 4 DISCUSSION

To our knowledge, the present study for the first time explored the relationship between functional network changes of NAc subregions and NMS phenotypes in PD. We identified that the left core, left shell, and right core had reduced FC with extensive brain regions, and each subregion had special PD‐related FC changes (Figure 2). Functional network changes in the left core, left shell, and right core were commonly associated with the presence of depression, anxiety, apathy, and cognitive impairment. Moreover, the left core and left shell were associated with excessive daytime sleepiness, whereas the right core was associated with olfactory impairment and RBD (Figure 4D).

We found that the left NAc was more damaged in PD, as both the left shell and core showed reduced FC, while there were no significant FC changes in the right shell. This phenomenon may be due to the asymmetric degeneration of the central neural system. As most of our PD patients (61%) were right‐side onset, the left hemisphere was more affected in most patients. And PD patients with predominant right motor symptoms (left‐hemisphere damage) present more severe NMS.^20^

In PD patients, the left core, left shell, and right core commonly had reduced FC with extensive brain regions (Figure 2 and Supplementary Table S1). Among these regions, the attenuation of FC of circuits including NAc, superior temporal gyrus (BA22), OFC (BA11), right fusiform (BA37), right lingual gyrus, and right middle temporal gyrus (BA21) is closely related to major depressive disorder^21^, ^22^, ^23^, ^24^; while the left superior occipital gyrus (BA18),^25^ bilateral temporal poles (BA38), and left parahippocampal gyrus (BA36)^26^ are associated with emotion‐related problems, especially anxiety. The reduced FC of NAc with the left inferior frontal gyrus (BA47) and right insula (BA13) has been shown positively correlated with the severity of apathy in PD.^27^ And the middle temporal gyrus, hippocampus (BA36), parahippocampal gyrus, and left superior occipital gyrus are involved in memory processing.^28^, ^29^ The similarity of network changes helps to explain why these subregions are commonly related to the presence of depression, anxiety, apathy, and cognitive impairment.

In addition, we found that the left core and left shell both had reduced FC with the amygdala, hippocampus, and left middle occipital gyrus (BA19), whereas the bilateral core had decreased FC with the right inferior frontal gyrus (BA47), right Rolandic operculum, and right supramarginal gyrus (BA40) (Figure 2 and Supplementary Table S1). Neural input from the hippocampus to the shell involves spatial cue‐reward information processing, while the FC between the core and hippocampus is associated with discrete cue‐reward information processing.^30^ The neural input inhibition from the hippocampus to the shell is involved in the pathophysiology of depression,^31^ and the activity in NAc, amygdala, hippocampus, and ventromedial PFC (BA11) is associated with excessive daytime sleepiness.^32^, ^33^ EEG studies have shown that local field potential alternations in the basal ganglia are associated with depression in PD, while reduced intertemporal and frontotemporal FC is related to PD dementia.^34^

In addition to these commonalities, each subregion of NAc also exhibited specifically PD‐related network changes (Figure 2 and Supplementary Table S1). The right core had reduced FC with the precentral gyrus (BA6), superior frontal gyrus (BA6), parietal gyrus, and SMA (BA6), and dysfunction of these regions has been detected in RBD patients.^35^, ^36^, ^37^, ^38^ The right core also showed decreased FC with the left supramarginal gyrus (BA40), right middle temporal gyrus, left parietal lobe, and left OFC, whereas the left shell had reduced FC with the right OFC and right ventromedial PFC. Previous studies have demonstrated the progressive decrease of network connectivity including these regions is related to cognitive deficits in PD patients.^39^, ^40^ Additionally, the impairment of the inferior frontal gyrus and ventral striatum contributes to olfactory dysfunction in PD, and the UPSIT score is positively correlated with the regional cerebral blood flow in the right superior occipital gyrus.^36^, ^41^ These special network modulations might be a reason contributing to the different associations of each subregion with NMS phenotypes in PD.

The subregions of NAc were commonly associated with the presence of apathy, anxiety, depression, and cognitive impairment (Figure 4D). The inputs from the frontal/temporal lobe serve as feedback pathways to dictate particular response priorities, and NAc integrates information from frontal/temporal regions to regulate cognitive and affective processes.^4^ Intracranial EEG studies found that the hippocampus‐NAc loop promotes memory formation.^42^ The inputs from the PFC to NAc are related to cognitive functions, while the amygdala afferent to NAc may involve in the expression of emotion and affective conditioning processes.^43^ These findings link the dysfunction of NAc to major depressive disorder, anxiety, apathy, and cognitive impairment.

The dysfunction of neurotransmitters in the NAc is related to the NMS of PD, which may support the association between neural network changes in NAc and the NMS in PD. The loss of glutamate delta‐1 receptor leads to reduced inhibitory neurotransmitters of medium spiny neurons in the core of NAc and results in neuropsychiatric disorders, especially anxiety and depression‐like behaviors.^44^ The decreased levels of dopamine D2/D3 receptors in NAc may account for the apathy in PD.^45^ The dysfunction of adenosine A_2A_ receptors in NAc is relevant to cognitive impairment via modulating dopamine and glutamate homeostasis in PD.^46^ A_2A_ receptor antagonists can reverse cognitive impairment and depression in experimental models of PD.^47^ We found that the imaging scores and behavioral scores are correlated (Figure 5), which indicates that PD patients with a greater reduction of network connectivity in NAc are more likely to have these NMS.

NAc medium spiny neurons expressing dopamine D1 (D1‐MSNs) or D2 receptors (D2‐MSNs) can regulate sleep‐related processing. The D1‐MSNs play roles in rapid eye movement sleep, whereas D2‐MSNs regulate slow‐wave sleep.^48^ GABAergic neurons of the NAc may induce sleep via their inhibitory projections to the waking systems such as the hypothalamic hypocretin and histaminergic neurons, the ventral tegmental dopaminergic and the noradrenergic locus coeruleus neurons.^49^ The arousal effects of caffeine have been attributed in part to its effects on A_2A_ receptors localized to the shell of the NAc.^50^ Istradefylline, a selective adenosine A_2A_ receptor antagonist, can improve daytime sleepiness without impairing nocturnal sleep.^51^ Thus, the neuromodulation imbalance of the left shell and core is likely important to the onset of excessive daytime sleepiness.

The olfactory deficit is caused by dopaminergic denervation to the NAc and olfactory tubercle according to animal studies, as dopamine receptor modulation of olfactory performance may be acting inside the NAc and olfactory tubercle, and dopaminergic denervation could induce olfactory impairment.^52^ The D1‐MSNs in the NAc core release the excitatory peptide substance P,^53^ while substance P is involved in the regulation of sensory processes such as olfaction.^54^ As discussed above, the NAc is involved in sleep‐related processing. A multimodal imaging study found lower network strength of the NAc in RBD patients compared with HC.^55^

In addition to NMS, neural network changes in NAc are related to some other features, such as age, education, UPDRS‐III, H&Y, LEDD, gray matter volume, and the volume of the NAc subregions (Figures S3‐S5). It is well known that these features can influence functional network connectivity.^56^, ^57^ PD is an age‐related disease, and education, antiparkinsonian medication, disease duration, and severity are important factors contributing to NMS.^2^ Although we did not find a significant difference in the volume of NAc in PD patients compared with HC, previous studies have shown that decreased volume of NAc is associated with cognitive impairment and apathy in PD.^12^, ^13^ Therefore, we removed these features in our PLS analysis to eliminate the interactions between these features and neural network alterations and to fully explore the associations between FC changes and NMS phenotypes.

This study has some limitations. We did not find a significant association between impulsive–compulsive disorders (ICD) and network changes of NAc, which is likely due to the small number of our PD patients presented with ICD (12 ICD in 116 PD patients). The incidence of ICD in PD is only 4.15% in Chinese people.^58^ And PD patients with more than 1000 mg of LEDD are more likely to suffer from ICD,^59^ while a relatively low dosage of antiparkinsonian medications was used in our patients (average LEDD was 312 mg). Second, this study is a cross‐sectional study, future studies are needed to investigate longitudinal neural network alterations in PD and how they relate to the progression of NMS.

## 5 CONCLUSION

This study identified the decreased FC in the left core, left shell, and right core in PD patients. FC changes in these three subregions are commonly associated with depression, anxiety, apathy, and cognitive impairment. Moreover, the left shell and core are associated with excessive daytime sleepiness, whereas the right core is associated with olfactory impairment and RBD. Our findings for the first time reveal the association between the NAc subregions and the NMS phenotypes and provide new insights into the pathogenesis of NMS in PD.

## AUTHOR CONTRIBUTIONS

LC analyzed the data and wrote the manuscript. JW guided the analyses of MRI data and PLS correlation analysis. MX and LS guided the PLS analysis. JS, LG, and DZ collected clinical and MRI data. TW designed and supervised the whole research and polished the manuscript. All authors read and approved the final manuscript.

## CONFLICT OF INTEREST

All authors declare that they have no conflict of interest.

## Supporting information


Figure S1

Figure S2

Figure S3

Figure S4

Figure S5

Table S1‐S2

Table S3

Data S1

## Data Availability

The data sets or codes used and/or analyzed during the current study are available from the corresponding author upon reasonable request.
