# Supplementary material for: Altered functional connectivity of nucleus accumbens subregions associates with non‐motor symptoms in Parkinson's disease
Source: CNS Neurosci Ther. 2022 Oct 2;28(12):2308–18. doi: 10.1111/cns.13979 (PMC9627369; doi:10.1111/cns.13979)
Supplement: Supplementary file 1 — Figure S1 [file CNS-28-2308-s007.pdf]

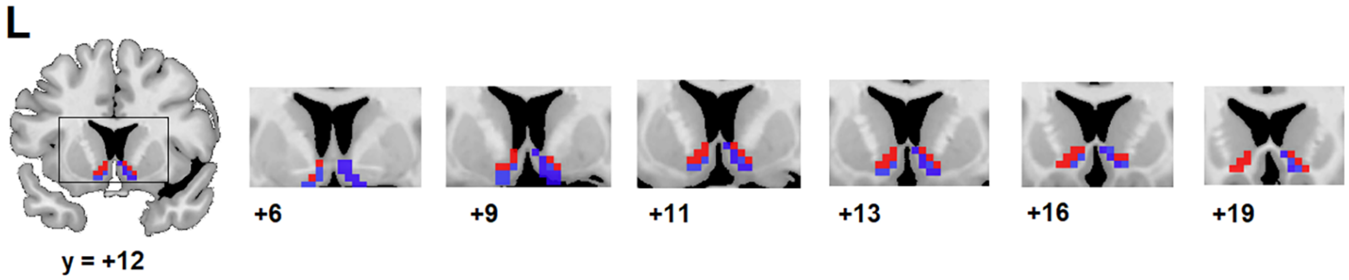

Fig. S1 ROIs in the coronal sections.
Red regions represent the core, and blue regions represent the shell of the nucleus accumbens. We chose bilateral core and shell as the ROIs (ROI1-left core, ROI2-left shell, ROI3-right core, ROI4-right shell). ROIs: regions of interest
