# Supplementary material for: Altered functional connectivity of nucleus accumbens subregions associates with non‐motor symptoms in Parkinson's disease
Source: CNS Neurosci Ther. 2022 Oct 2;28(12):2308–18. doi: 10.1111/cns.13979 (PMC9627369; doi:10.1111/cns.13979)
Supplement: Supplementary file 2 — Figure S2 [file CNS-28-2308-s008.pdf]

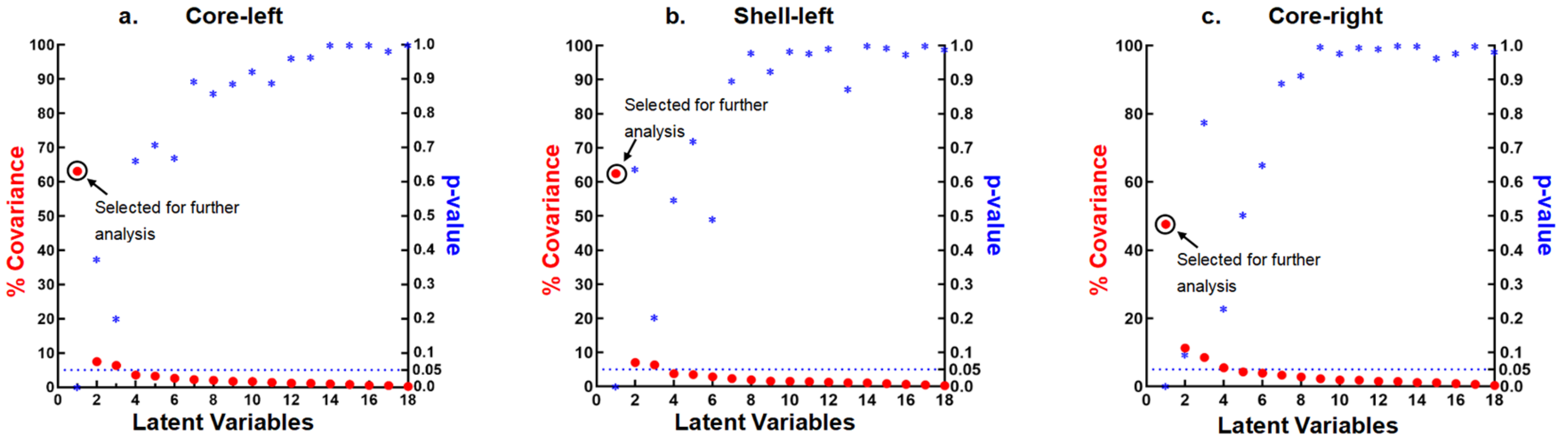

Fig. S2 Covariance explained and permutation p-values.

Covariance explained and permutation p-values for all latent variables in the PLS analysis in the left core (a), left shell (b), and right core (c). The first latent variable (LV-I) is selected for further analysis.
