# Supplementary material for: Altered functional connectivity of nucleus accumbens subregions associates with non‐motor symptoms in Parkinson's disease
Source: CNS Neurosci Ther. 2022 Oct 2;28(12):2308–18. doi: 10.1111/cns.13979 (PMC9627369; doi:10.1111/cns.13979)
Supplement: Supplementary file 5 — Figure S5 [file CNS-28-2308-s006.pdf]

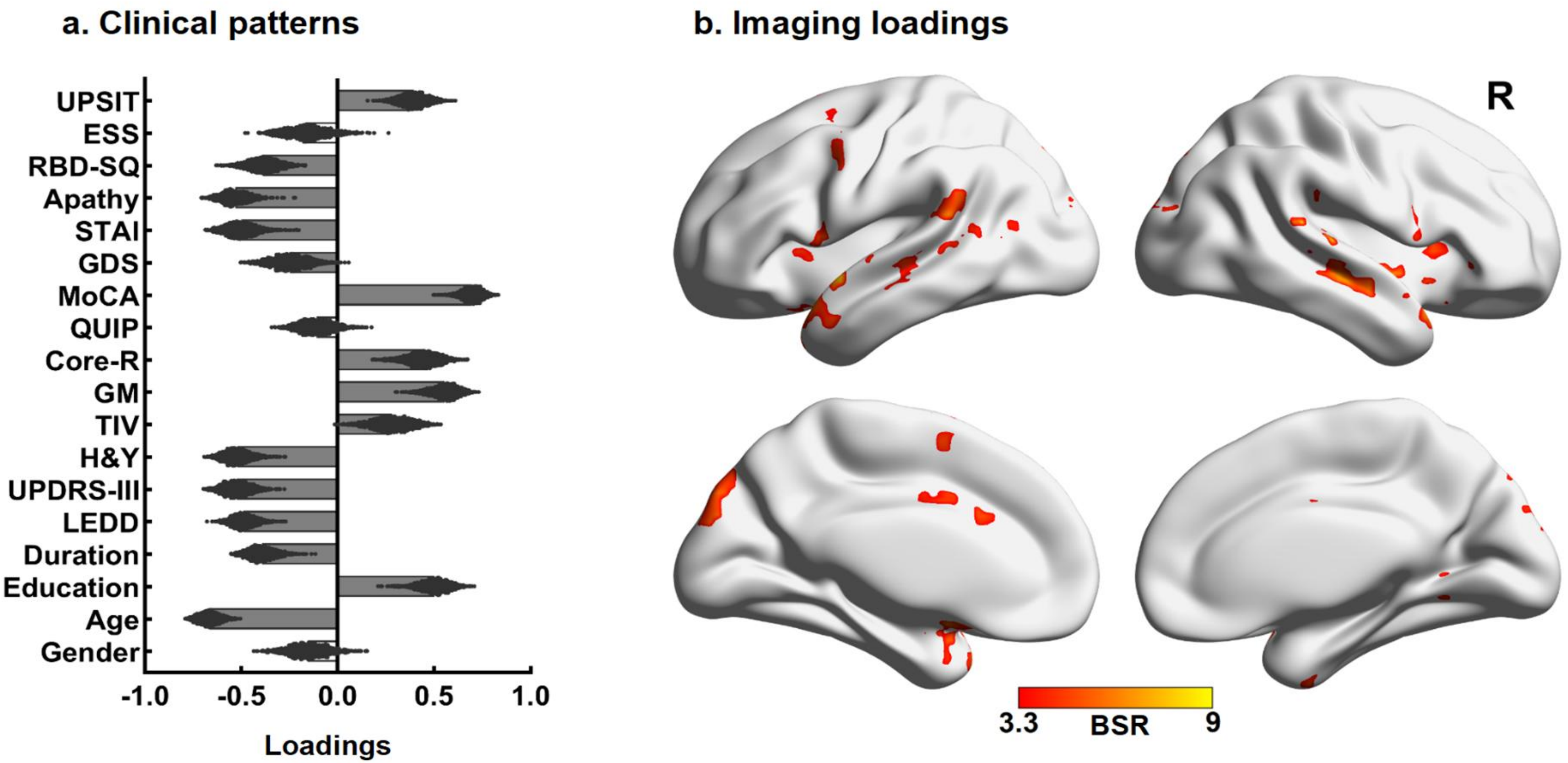

Fig. S5 The FC-behavior pattern in PLS analysis results of the right core.
a) Clinical behavioral pattern. Grey bars represent variables associated with the pattern.White bars represent no significant. b) Brain imaging pattern bootstrap ratios in MNI space. PLS, partial least squares; UPSIT, University of Pennsylvania Smell Identification Test; ESS, Epworth Sleepiness Scale; RBD-SQ, REM Sleep Behavior Disorder Screening Questionnaire; Apathy, Movement Disorder Society Unified Parkinson' s Disease Rating Scale, part I, Question for apathy; STAI, State-Trait Anxiety Inventory, T-AI part; GDS, Geriatric Depression Scale-15; MoCA, Montreal Cognitive Assessment; QUIP, Questionnaire for Impulsive-Compulsive Disorders in Parkinson' s Disease; Core-R, right core volume; GM, grey matter volume; TIV, total intracranial volume; H&Y, Hoehn and Yahr disability scale; UPDRS-III, Movement Disorder Society Unified Parkinson' s Disease Rating Scale, part III; LEDD, Levodopa equivalent daily dose; Duration, disease duration.
