# Supplementary material for: Altered functional connectivity of nucleus accumbens subregions associates with non‐motor symptoms in Parkinson's disease
Source: CNS Neurosci Ther. 2022 Oct 2;28(12):2308–18. doi: 10.1111/cns.13979 (PMC9627369; doi:10.1111/cns.13979)
Supplement: Supplementary file 6 — Table S1‐S2 [file CNS-28-2308-s005.docx]

Supplementary Table 1. Brain regions showing reduced functional connectivity with the ROIs

| ROIs | Hemi-cerebrum | Cluster size >10 | Regions (AAL3) | Brodmann | | |  | MNI | |  | Peak _T_ | |
| --- | --- | --- | --- | --- | --- | --- | --- | --- | --- | --- | --- | --- |
|  |  |  |  |  |  |  | x, y, z {mm} | | | |  |  |
| Core-left | left | 184 | Temporal_Mid_L**^***^** | 21 | | -45 | | -51 | 6 | | 5.32 | |
|  |  | 184 | Temporal_Sup_L**^***^** | 22 | | -54 | | 3 | -9 | | 5.29 | |
|  |  | 103 | Temporal_Pole_Sup_L**^***^** | | 38 | -54 | | 6 | -9 | | 4.52 | |
|  |  | 42 | Occipital_Mid_L^△^ | 19 | | -39 | | -75 | 12 | | 4.00 | |
|  |  | 36 | Cuneus_L**^***^** |  | | -6 | | -93 | 30 | | 4.53 | |
|  |  | 34 | ParaHippocampal_L^△^ | 36 | | -21 | | -36 | -9 | | 4.53 | |
|  |  | 30 | Hippocampus_L^△^ | 36 | | -18 | | -33 | -6 | | 4.26 | |
|  |  | 30 | Amygdala_L^△^ |  | | -24 | | 0 | -24 | | | 4.53 |
|  |  | 24 | Occipital_Sup_L**^***^** | 18 | | -15 | | -84 | 24 | | 4.20 | |
|  |  | 17 | ACC_sup_L^▲^ | 32 | | -6 | | 21 | 24 | | 4.34 | |
|  |  | 17 | Olfactory_L**^***^** | 34 | | -18 | | 9 | -18 | | | 3.68 |
|  |  | 14 | Frontal_Inf_Oper_L**^***^** | 47 | | -51 | | 12 | 3 | | 3.45 | |
|  | right | 211 | Temporal_Sup_R**^***^** | 22 | | 45 | | -9 | -6 | | 5.51 | |
|  |  | 125 | Temporal_Pole_Sup_R**^***^** | | 38 | 45 | | 12 | -21 | | | 6.20 |
|  |  | 87 | Insula_R**^***^** | 16 | | 45 | | -6 | -6 | | 5.28 | |
|  |  | 43 | Fusiform_R**^***^** | 37 | | 36 | | 0 | -36 | | | 4.64 |
|  |  | 33 | Amygdala_R^△^ |  | | 24 | | 0 | -21 | | | 5.08 |
|  |  | 29 | ParaHippocampal_R**^***^** | 36 | | 21 | | 0 | -21 | | | 4.82 |
|  |  | 27 | Precentral_R^*^ | 6 | | 51 | | 9 | 42 | | 3.89 | |
|  |  | 22 | Lingual_R**^***^** |  | | 24 | | -54 | -3 | | 3.85 | |
|  |  | 21 | Heschl_R**^***^** | 48 | | 48 | | -12 | 6 | | 4.25 | |
|  |  | 18 | Rolandic_Oper_R^▲^ |  | | 51 | | 6 | 9 | | 3.48 | |
|  |  | 16 | SupraMarginal_R^▲^ | 40 | | 69 | | -27 | 18 | | 3.93 | |
|  |  | 16 | Frontal_Inf_Orb_R^▲^ | 47 | | 33 | | 30 | -6 | | 3.84 | |
|  |  | 14 | Frontal_Inf_Oper_R^▲^ | 47 | | 51 | | 9 | 6 | | 3.30 | |
| Shell-left | left | 209 | Temporal_Mid_L**^***^** | 21 | | -48 | | -15 | -12 | | | 4.81 |
|  |  | 191 | Temporal_Sup_L**^***^** | 22 | | -54 | | 3 | -9 | | 5.17 | |
|  |  | 137 | Temporal_Pole_Sup_L**^***^** | | 38 | -24 | | 6 | -27 | | | 4.98 |
|  |  | 70 | ParaHippocampal_L^△^ | 36 | | -24 | | 3 | -27 | | | 4.94 |
|  |  | 62 | Hippocampus_L^△^ | 36 | | -21 | | -6 | -21 | | | 4.69 |
|  |  | 58 | Temporal_Pole_Mid_L^▼^ | 38 | | -48 | | 15 | -27 | | | 4.62 |
|  |  | 43 | Fusiform_L^*^ | 37 | | -36 | | -24 | -27 | | | 3.76 |
|  |  | 38 | Amygdala_L^△^ |  | | -24 | | 0 | -24 | | | 5.38 |
|  |  | 34 | Temporal_Inf_L^*^ | 20 | | -45 | | -6 | -27 | | | 5.21 |
|  |  | 29 | Occipital_Mid_L^△^ | 19 | | -39 | | -78 | 3 | | 3.89 | |
|  |  | 21 | OFCmed_L^▼^ | 11 | | -12 | | 45 | -21 | | | 4.04 |
|  |  | 19 | Cuneus_L**^***^** |  | | -6 | | -93 | 27 | | 3.55 | |
|  |  | 17 | Cingulum_Mid_L^▼^ | 24 | | -6 | | 6 | 33 | | 3.30 | |
|  |  | 16 | Occipital_Sup_L**^***^** | 18 | | -15 | | -84 | 24 | | 3.94 | |
|  |  | 15 | Rectus_L^▼^ | 11 | | 3 | | 33 | -18 | | | 4.13 |
|  |  | 14 | Frontal_Inf_Oper_L**^***^** | 47 | | -51 | | 12 | 3 | | 3.60 | |
|  |  | 12 | Olfactory_L**^***^** | 34 | | -18 | | 9 | -18 | | | 3.84 |
|  | right | 209 | Temporal_Sup_R**^***^** | 22 | | 45 | | -9 | -9 | | 5.59 | |
|  |  | 100 | Temporal_Pole_Sup_R**^***^** | | 38 | 45 | | 12 | -21 | | | 6.36 |
|  |  | 98 | ParaHippocampal_R**^***^** | 36 | | 21 | | -3 | -21 | | | 5.05 |
|  |  | 78 | Insula_R**^***^** | 16 | | 45 | | -6 | -6 | | 5.10 | |
|  |  | 41 | OFCpost_R^*^ | 11 | | 24 | | 21 | -21 | | | 3.88 |
|  |  | 36 | Amygdala_R^△^ |  | | 24 | | 0 | -21 | | | 5.27 |
|  |  | 34 | Fusiform_R**^***^** | 37 | | 36 | | 0 | -36 | | | 5.40 |
|  |  | 33 | Hippocampus_R^*^ | 37 | | 27 | | -15 | -21 | | | 5.27 |
|  |  | 30 | Temporal_Pole_Mid_R^▼^ | 38 | | 39 | | 0 | -36 | | | 4.76 |
|  |  | 26 | Heschl_R**^***^** | 48 | | 42 | | -24 | 6 | | 4.42 | |
|  |  | 24 | OFCmed_R^*^ | 11 | | 18 | | 27 | -16 | | | 4.32 |
|  |  | 21 | Lingual_R**^***^** |  | | 15 | | -51 | -6 | | 3.93 | |
|  |  | 20 | Occipital_Mid_R^*^ | 19 | | 39 | | -84 | 9 | | 3.43 | |
|  |  | 17 | Frontal_Med_Orb_R^*^ | 11 | | 3 | | 36 | -12 | | | 3.77 |
|  |  | 15 | Temporal_Inf_R | 20 | | 36 | | 0 | -39 | | | 4.32 |
|  |  | 14 | Rectus_R^*^ | 11 | | 3 | | 33 | -18 | | | 4.13 |
| Core-right | left | 324 | Temporal_Mid_L**^***^** | 21 | | -54 | | -24 | -3 | | 5.28 | |
|  |  | 119 | Temporal_Sup_L**^***^** | 22 | | -66 | | -42 | 18 | | 5.03 | |
|  |  | 98 | Temporal_Pole_Sup_L**^***^** | | 38 | -48 | | 9 | -6 | | 4.51 | |
|  |  | 65 | Supp_Motor_Area_L^*^ | 6 | | -6 | | 6 | 66 | | 4.18 | |
|  |  | 64 | Occipital_Sup_L**^***^** | 18 | | -15 | | -84 | 24 | | 4.55 | |
|  |  | 61 | Precentral_L^*^ | 6 | | -45 | | 3 | 45 | | 3.85 | |
|  |  | 58 | SupraMarginal_L^*^ | 40 | | -57 | | -45 | 24 | | 4.17 | |
|  |  | 56 | Cuneus_L**^***^** |  | | -6 | | -90 | 27 | | 4.05 | |
|  |  | 48 | Temporal_Pole_Mid_L^▼^ | 38 | | -36 | | 15 | -33 | | | 4.21 |
|  |  | 47 | Insula_L^*^ | 16 | | -27 | | 18 | -18 | | | 5.21 |
|  |  | 45 | OFCpost_L^*^ | 11 | | -24 | | 18 | -18 | | | 5.21 |
|  |  | 41 | Frontal_Sup_L^*^ | 6 | | -12 | | 18 | 51 | | 4.40 | |
|  |  | 31 | Parietal_Inf_L^*^ |  | | -33 | | -48 | 39 | | 3.37 | |
|  |  | 30 | Frontal_Mid_L^*^ |  | | -27 | | 6 | 57 | | 3.61 | |
|  |  | 29 | OFCmed_L^▼^ | 11 | | -12 | | 21 | -21 | | | 3.79 |
|  |  | 28 | Olfactory_L**^***^** | 34 | | -18 | | 9 | -15 | | | 4.88 |
|  |  | 28 | Cingulum_Mid_L^▼^ | 24 | | 0 | | 6 | 33 | | 3.83 | |
|  |  | 26 | ACC_sup_L^▲^ | 32 | | 0 | | 18 | 27 | | 3.87 | |
|  |  | 18 | Frontal_Inf_Oper_L**^***^** | 47 | | -54 | | 9 | 6 | | 3.57 | |
|  |  | 17 | Parietal_Sup_L^*^ |  | | -15 | | -81 | 45 | | 3.73 | |
|  |  | 14 | Angular_L^*^ | 39 | | -42 | | -54 | 33 | | 3.50 | |
|  |  | 14 | Rectus_L^▼^ | 11 | | -18 | | 12 | -15 | | | 4.69 |
|  | right | 157 | Temporal_Sup_R**^***^** | 22 | | 54 | | -21 | -6 | | 4.97 | |
|  |  | 135 | Insula_R**^***^** | 13 | | 39 | | -6 | -6 | | 5.30 | |
|  |  | 67 | Temporal_Mid_R^*^ | 21 | | 54 | | -12 | -12 | | | 4.53 |
|  |  | 65 | SupraMarginal_R^▲^ | 40 | | 69 | | -36 | 24 | | 4.15 | |
|  |  | 56 | Occipital_Sup_R^*^ | 18 | | 21 | | -75 | 36 | | 3.68 | |
|  |  | 56 | Frontal_Inf_Oper_R^▲^ | 47 | | 60 | | 12 | 6 | | 4.27 | |
|  |  | 52 | Cuneus_R^*^ |  | | 15 | | -90 | 21 | | 3.69 | |
|  |  | 42 | Temporal_Pole_Sup_R**^***^** | | 38 | 48 | | 12 | -24 | | | 4.24 |
|  |  | 35 | Frontal_Inf_Orb_R^▲^ | 47 | | 39 | | 30 | -3 | | 3.63 | |
|  |  | 23 | Lingual_R**^***^** |  | | 21 | | -57 | -3 | | 4.11 | |
|  |  | 22 | Frontal_Inf_Tri_R^*^ | 47 | | 39 | | 30 | 0 | | 3.54 | |
|  |  | 22 | Temporal_Pole_Mid_R^▼^ | 38 | | 39 | | 12 | -36 | | | 3.71 |
|  |  | 20 | Fusiform_R**^***^** | 37 | | 27 | | -3 | -42 | | | 4.10 |
|  |  | 19 | Rolandic_Oper_R^▲^ |  | | 45 | | -24 | 15 | | 3.57 | |
|  |  | 18 | ParaHippocampal_R**^***^** | 36 | | 30 | | 0 | -33 | | | 3.93 |
|  |  | 12 | Heschl_R**^***^** | 48 | | 48 | | -12 | 6 | | 3.66 | |
|  |  | 12 | Calcarine_R^*^ |  | | 15 | | -93 | 12 | | 4.09 | |

^***^ Brain regions showing reduced functional connectivity with three subregions

* Brain regions only showing reduced functional connectivity with the corresponding subregion

^△^ Brain regions showing reduced functional connectivity with both left core and left shell

^▲^ Brain regions showing reduced functional connectivity with bilateral cores

^▼^ Brain regions showing reduced functional connectivity with left shell and right core

Supplementary Table 2 . Peak coordinates in MNI-ICBM152 space for imaging pattern using bootstrap ratios of PLS results with covariates.

| ROIs | Cluster size  >10 | Brain regions (AAL3) | Brodmann | MNI (Peak coordinate) | | | Peak B-ratios |
| --- | --- | --- | --- | --- | --- | --- | --- |
|  |  |  |  | x | y | z |  |
| Core-left | 204 | Temporal_Sup_R | 22 | 60 | 0 | -9 | 12.36 |
|  | 167 | Temporal_Sup_L | 22 | -48 | -18 | 0 | 13.29 |
|  | 154 | Temporal_Mid_L | 21 | -60 | -39 | 3 | 11.16 |
|  | 93 | Temporal_Pole_Sup_R | 38 | 60 | 3 | -9 | 12.17 |
|  | 92 | Temporal_Pole_Sup_L | 38 | -48 | 15 | -18 | 8.04 |
|  | 65 | Insula_R | 16 | 39 | -15 | 12 | 9.58 |
|  | 41 | Occipital_Mid_L | 19 | -45 | -81 | 15 | 8.93 |
|  | 33 | Amygdala_R |  | 30 | -3 | -15 | 8.52 |
|  | 30 | Cuneus_L |  | -6 | -87 | 33 | 8.74 |
|  | 24 | Precentral_R | 6 | 54 | 6 | 36 | 9.68 |
|  | 24 | Lingual_R |  | 21 | -54 | -3 | 7.58 |
|  | 24 | Fusiform_R | 37 | 36 | -3 | -36 | 7.05 |
|  | 23 | ParaHippocampal_R | 36 | 33 | -3 | -30 | 7.96 |
|  | 21 | ParaHippocampal_L | 36 | -27 | -27 | -18 | 6.95 |
|  | 19 | Occipital_Sup_L | 18 | -12 | -87 | 21 | 6.95 |
|  | 19 | Amygdala_L |  | -24 | -6 | -18 | 6.66 |
|  | 18 | Heschl_R | 48 | 51 | -12 | 6 | 10.62 |
|  | 18 | SupraMarginal_R | 40 | 66 | -27 | 18 | 7.58 |
|  | 16 | Hippocampus_L | 36 | -24 | -6 | -21 | 6.43 |
|  | 17 | Olfactory_L | 34 | -9 | 15 | -18 | 6.32 |
|  | 16 | Rolandic_Oper_R |  | 48 | -15 | 9 | 9.75 |
|  | 16 | Frontal_Inf_Oper_R | 47 | 48 | 15 | 12 | 6.59 |
|  | 14 | Frontal_Inf_Oper_L | 47 | -51 | 9 | 3 | 9.06 |
|  | 11 | Frontal_Inf_Orb_R | 47 | 33 | 27 | -6 | 6.52 |
|  | 10 | Frontal_Mid_R |  | 45 | 6 | 39 | 6.62 |
| Shell-left | 187 | Temporal_Mid_L | 21 | -57 | -18 | 0 | 11.75 |
|  | 184 | Temporal_Sup_R | 22 | 60 | 0 | -9 | 11.61 |
|  | 177 | Temporal_Sup_L | 22 | -54 | -21 | 3 | 12.74 |
|  | 108 | Temporal_Pole_Sup_L | 38 | -54 | 6 | -12 | 7.90 |
|  | 85 | Temporal_Pole_Sup_R | 38 | 60 | 3 | -9 | 10.74 |
|  | 81 | ParaHippocampal_R | 36 | 30 | -27 | -18 | 8.38 |
|  | 40 | Hippocampus_R | 36 | 24 | -3 | -24 | 7.56 |
|  | 39 | ParaHippocampal_L | 30 | -27 | -27 | -18 | 7.43 |
|  | 38 | Amygdala_R |  | 27 | 0 | -18 | 10.24 |
|  | 37 | Insula_R | 48 | 39 | -6 | -6 | 7.05 |
|  | 35 | Hippocampus_L | 36 | -24 | -6 | -24 | 8.36 |
|  | 33 | Temporal_Pole_Mid_L | 38 | -45 | 12 | -27 | 6.76 |
|  | 28 | Occipital_Mid_L | 19 | -42 | -78 | 0 | 5.84 |
|  | 26 | Temporal_Pole_Mid_R | 38 | 51 | 3 | -18 | 8.83 |
|  | 26 | Amygdala_L |  | -30 | -6 | -15 | 7.79 |
|  | 24 | Heschl_R | 48 | 48 | -21 | 6 | 7.93 |
|  | 24 | Fusiform_R | 37 | 30 | -30 | -21 | 7.82 |
|  | 20 | Lingual_R |  | 18 | -45 | -6 | 5.94 |
|  | 16 | Cuneus_L |  | -6 | -87 | 33 | 9.14 |
|  | 16 | Frontal_Med_Orb_R | 11 | 0 | 30 | -12 | 7.21 |
|  | 15 | Occipital_Sup_L | 18 | -12 | -87 | 18 | 7.16 |
|  | 14 | OFCpost_R | 11 | 24 | 18 | -21 | 5.77 |
|  | 12 | Frontal_Inf_Oper_L | 47 | -57 | 6 | 9 | 8.11 |
|  | 12 | Olfactory_L | 34 | -21 | 6 | -15 | 7.26 |
|  | 10 | Temporal_Inf_L | 20 | -57 | -24 | -18 | 6.51 |
|  | 10 | Frontal_Med_Orb_L | 11 | 3 | 30 | -15 | 6.46 |
| Core-right | 122 | Temporal_Sup_R | 22 | 66 | -12 | 3 | 8.26 |
|  | 100 | Temporal_Mid_L | 21 | -63 | -12 | -6 | 6.32 |
|  | 97 | Temporal_Sup_L | 22 | -60 | -9 | 0 | 7.49 |
|  | 62 | Cuneus_R |  | 15 | -96 | 12 | 8.95 |
|  | 59 | Occipital_Sup_L | 18 | -18 | -75 | 39 | 8.29 |
|  | 58 | Temporal_Pole_Sup_L | 38 | -54 | 15 | -12 | 6.80 |
|  | 54 | SupraMarginal_R | 40 | 63 | -18 | 21 | 6.90 |
|  | 52 | SupraMarginal_L | 40 | -60 | -36 | 24 | 5.96 |
|  | 52 | Precentral_L | 6 | -48 | 0 | 39 | 5.76 |
|  | 46 | Cuneus_L |  | -15 | -84 | 36 | 7.15 |
|  | 43 | Frontal_Inf_Oper_R | 47 | 57 | 9 | 3 | 6.71 |
|  | 37 | Occipital_Sup_R | 18 | 21 | -81 | 18 | 7.76 |
|  | 29 | Temporal_Pole_Sup_R | 38 | 54 | 6 | -15 | 6.17 |
|  | 27 | Parietal_Inf_L |  | -33 | -42 | 42 | 4.86 |
|  | 22 | Lingual_R |  | 21 | -60 | -6 | 6.93 |
|  | 21 | Supp_Motor_Area_L | 6 | -3 | 3 | 54 | 6.78 |
|  | 20 | Insula_R | 48 | 39 | -15 | 12 | 6.92 |
|  | 20 | Cingulate_Mid_L | 24 | -6 | 9 | 33 | 4.83 |
|  | 19 | Parietal_Sup_L |  | -18 | -72 | 39 | 8.07 |
|  | 18 | Rolandic_Oper_R |  | 51 | -9 | 9 | 7.53 |
|  | 18 | Frontal_Sup_L | 6 | -21 | 0 | 63 | 4.58 |
|  | 17 | Temporal_Mid_R | 21 | 54 | -24 | -6 | 4.42 |
|  | 16 | Frontal_Inf_Oper_L | 47 | -54 | 6 | 12 | 5.43 |
|  | 15 | Calcarine_R |  | 15 | -93 | 9 | 9.40 |
|  | 14 | Olfactory_L | 34 | -21 | 6 | -15 | 5.11 |

PLS, partial least squares; B-ratios, bootstrap ratio.

Brain regions are ordered within the subregions by cluster size.
