# Supplementary material for: Altered functional connectivity of nucleus accumbens subregions associates with non‐motor symptoms in Parkinson's disease
Source: CNS Neurosci Ther. 2022 Oct 2;28(12):2308–18. doi: 10.1111/cns.13979 (PMC9627369; doi:10.1111/cns.13979)
Supplement: Supplementary file 7 — Table S3 [file CNS-28-2308-s002.docx]

Supplementary Table 3. Demographic and clinical features of all participants

|  | PD (mean ± SD） | HC (mean ± SD） |
| --- | --- | --- |
| Database 1 |  |  |
| Age (years) | 59.43 ± 10.77 | 65.20 ± 8.13 |
| Gender (male/female) | 28/14 | 13/2 |
| Duration (years) | 1.50 ± 1.14 | NA |
| LEDD (mg) | 105.71± 247.71 | NA |
| UPDRS-III | 22.88 ± 12.04 | NA |
| H&Y Stage | 1.76 ± 0.48 | NA |
| QUIP | 0.10 ± 0.30 | 0 |
| MoCA | 27.24 ± 2.94 | 27.87 ± 1.41 |
| GDS | 2.12 ± 2.48 | 1.2 ± 3.57 |
| STAI | 32.50 ± 10.93 | 28.73 ±5.48 |
| UPDRS-I-Apathy | 0.24 ± 0.58 | 0 |
| RBD-SQ | 5.02 ± 2.78 | 3.80 ± 1.66 |
| Epworth | 5.83 ± 3.79 | 5.73 ± 2.91 |
| UPSIT | 24.24 ± 9.57 | 34.93 ± 3.15 |
| Database 2 |  |  |
| Age (years) | 59.90 ± 9.05 | 59.84 ± 8.48 |
| Gender (male/female) | 41/46 | 38/53 |
| Duration (years) | 4.49 ± 3.95 | NA |
| LEDD (mg) | 381.78 ± 287.69 | NA |
| UPDRS-III | 27.17 ± 12.03 | NA |
| H&Y Stage | 1.71 ± 0.61 | NA |
| QUIP | 0.10 ± 0.31 | 0 |
| MoCA | 23.40 ± 3.96 | 25.84 ± 2.78 |
| GDS | 2.94 ± 2.38 | 0 |
| STAI | 36.98 ± 10.27 | 27.78 ± 4.09 |
| UPDRS-I-Apathy | 0.66 ± 0.83 | 0.02 ± 0.15 |
| RBD-SQ | 4.75 ± 2.69 | 3.76 ± 1.91 |
| Epworth | 5.56 ± 4.80 | 3.82 ± 2.33 |
| UPSIT | 24.75 ± 9.26 | 34.42 ± 3.48 |

LEDD, Levodopa equivalent daily dose; UPDRS-III, Movement Disorder Society Unified Parkinson’s Disease Rating Scale, part III; QUIP, Questionnaire for Impulsive-Compulsive Disorders in Parkinson’s Disease; MoCA, Montreal Cognitive Assessment; GDS, Geriatric Depression Scale-15; STAI, State-Trait Anxiety Inventory, T-AI part; UPDRS-I-Apathy, Movement Disorder Society Unified Parkinson’s Disease Rating Scale, part I, Question for apathy; RBD-SQ, REM Sleep Behavior Disorder Screening Questionnaire; UPSIT, University of Pennsylvania Smell Identification Test.

Database 1 was from the PPMI (Parkinson’s Progression Markers Initiative) database ([www.ppmi-info.org/data](http://www.ppmi-info.org/data)). Database 2 was from the Movement Disorders Center of the Tiantan Hospital of Capital Medical University.
