# Supplementary material for: Altered functional connectivity of nucleus accumbens subregions associates with non‐motor symptoms in Parkinson's disease
Source: CNS Neurosci Ther. 2022 Oct 2;28(12):2308–18. doi: 10.1111/cns.13979 (PMC9627369; doi:10.1111/cns.13979)
Supplement: Supplementary file 8 — Data S1 [file CNS-28-2308-s004.docx]

**Supplementary Materials and methods**

**Participants**

This study included 129 PD patients and 106 age- and sex-matched healthy controls (HC). The diagnosis of PD was according to the MDS Clinical Diagnostic Criteria^1^. Of these subjects, 91 PD and 87 HC were from the Movement Disorders Center of the Tiantan Hospital of Capital Medical University, and 42 PD and 15 HC were from the PPMI (Parkinson’s Progression Markers Initiative) database (www.ppmi-info.org/data). The exclusion criteria for HC included: a history of neurological or psychiatric disorders, a family history of movement disorders, and obvious cerebral lesions. All subjects were assessed with the Montreal Cognitive Assessment (MoCA)^2^, Geriatric Depression Scale-15 (GDS)^3^, State-Trait Anxiety Inventory (STAI)^4^, Questionnaire for Impulsive-Compulsive Disorders in Parkinson’s Disease (QUIP)^5^, REM Sleep Behavior Disorder Screening Questionnaire (RBD-SQ)^6^, Epworth Sleepiness Scale (ESS)^7^, and University of Pennsylvania Smell Identification Test (UPSIT)^8^, while PD patients were additionally assessed with the MDS Unified Parkinson’s Disease Rating Scale (MDS-UPDRS)^9^, and the Hoehn and Yahr (H&Y) disability scale^10^. In addition, the levodopa-equivalent daily dose (LEDD) was calculated^11^. This study was conducted following the Declaration of Helsinki and was approved by the Institutional Review Board of Tiantan Hospital. All participants gave written informed consent before the study.

**MRI data acquisition**

MRI data were acquired using a 3T Magnetom Skyra scanner (Siemens, Erlangen, Germany) using a 20-channel head coil. PD patients were scanned in the “OFF” state (12 h withdrawal of medication). All participants were informed to keep their eyes closed, heads still, and not to think of anything. The 3D T1-weighted structural images were acquired with the following parameters: repetition time (TR) = 2530 ms, echo time (TE) = 2.98 ms, slice thickness = 1 mm, flip angle = 7°, field of view = 224 × 256 mm^2^, 192 sagittal slices. Resting-state Blood oxygen level-dependent images were acquired using gradient-echo echo-planar sequences with the following parameters: TR = 2,000 ms, TE = 30 ms, slice thickness = 3 mm, flip angle = 90°, field of view = 220 ×220 mm^2^, image matrix size = 64 × 64, 35 axial slices and the scanning time was 6 minutes.

PPMI data were collected on 3T Siemens scanners. T1-weighted structure image was also included with TR = 2300ms, TE = 2.98ms, 176 sagittal slices, slice thickness = 1 mm, flip angle = 9°, field of view = 240 × 256 mm^2^. Resting-state functional MRI (fMRI) data were obtained with the following parameters: TR = 2400ms, TE = 25ms, slice thickness = 3.3 mm, flip angle = 80°, field of view = 240 × 240 mm^2^, matrix size = 68 ×66, 40 axial slices and the total scanning time lasted 7 minutes.

**Preprocessing of imaging data**

Data were preprocessed using the Statistical Parametric Mapping (SPM12) and Data Processing and Analysis for Brain Imaging (DPABI) toolboxes based on MATLAB^12^. All images were transformed from DICOM format to nifti format file. The preprocessing steps of resting-state fMRI data were as follows: removing the first 10 time points; slice timing; head motion correction; coregistration of the structural image to functional image; structural image segmentation by DARTEL; spatial normalization into the standard Montreal Neurological Institute (MNI) brain template; spatial smoothing with a 4 mm full width at half maximum Gaussian smoothing kernel; nuisance covariates regression including cerebrospinal fluid signal, white matter signal, and 24-Friston head motion parameters; and temporal filter with 0.01~0.08 Hz. We defined the excessive head motion as more than 3 mm maximum translation in the x, y, or z plane, or 3° of maximum rotation around these three axes, and evaluated the mean framewise displacement (FD) parameters to control the head motion^13^.

**Regions of Interest (ROIs)**

We chose the bilateral core and shell of NAc as the ROIs (Fig. S1). The core and shell were defined based on a probabilistic atlas of NAc subregions^14^, which was created with 245 HC’s MRI images from the Human Connectome Project.

**Functional connectivity analysis**

Because the data were obtained from 9 different centers (one from Beijing and eight from PPMI), the ComBat model was used to eliminate center/scanner effects after the preprocessing steps, which is an empirical Bayes-based multivariate linear mixed-effects regression (github.com/Jfortin1/ComBatHarmonization)^15^. The four ROIs (bilateral core and shell of NAc) were used as the seeds for functional connectivity (FC) analysis. We obtained reference time courses via calculating the average time course of each ROI. Correlation analysis was carried out via calculating the temporal correlation between the seed reference and the whole brain in a voxel-wise manner. Then the individual correlation coefficient (*r*) maps were transformed into *z* maps using Fisher *r* to *z* transformation.

**Statistical Analyses**

IBM SPSS Statistics 25 software was used for statistical analysis of demographic and clinical information. Chi-square (χ^2^ ) test was performed to evaluate the statistical significance of gender. Kolmogorov-Smirnov-test was used to check the distribution normality of all data and the Mann-Whitney U test and two-sample t-tests were applied to measure the between-group difference.

For FC analysis, a second-level analysis of z maps was performed using SPM12. Two-sample T-tests were implemented to identify the FC differences between PD and HC in each ROI, with gender, age, education, and head motion as covariates. The significance of group differences was set at p < 0.05 with a false discovery rate (FDR) corrected. The extent threshold was 20 voxels. The results of the FC analysis were used for the subsequent PLS analysis.

**Measurement of the ROIs volumes**

We used the Computational Anatomy Toolbox for SPM (CAT12, <http://www.neuro.uni-jena.de/cat/>) for the structural images. Each original T1 image was spatially normalized by the DARTEL Toolbox and segmented into grey and white matter and cerebrospinal fluid. Then the total intracranial volume (TIV) and volume of grey matter were estimated. The volume of each ROI was measured by Image Calculator, a toolbox of DPABI. Finally, ANCOVA was used to measure the volume difference between PD and HC groups with covariates of age, gender and education.

**PLS analysis**

PLS correlation was used to analyze the association patterns between the PD-related FC network of each NAc subregion and the clinical features (Fig.1)^16^. The PD-related FC network based on the two-sample t-tests results of each NAc subregion was stored in matrix X_0_, while the demographic and clinical information was stored in matrix Y_0_. The matrices X_0_ and Y_0_ were normalized to matrices X and Y. Next, the correlation matrix R between the X and Y was computed. The matrix R was then subjected to singular value decomposition (R = VUS). The left (V) and right (U) singular vectors described the weights of every original voxel and every original clinical feature that contributed to each latent variable (LV), respectively. The covariance between the matrices V and U consisted of the matrix S. The number of LVs depends on the smallest dimension of these three constituent matrices. Here, the number of LVs was the number of clinical features (n = 18).

The permutation test was used to evaluate the statistical significance^17^. Briefly, the rows of X and Y were randomly permuted (N=1,000 repetitions), and a set of “null” correlation matrices were computed for singular value decomposition. We estimate the probability distribution and the significance was set as p-value below 0.05. Then, we selected the LVs with statistical significance for subsequent analysis. We projected the weighted patterns U and V onto individual-patient data to obtain the scalar imaging score and behavioral score:

imaging score, L_x_ = V X

behavioral score, L_y_ = U Y

The imaging score indicates the degree to which the brain FC change is expressed by an individual patient, and the behavioral score indicates the degree to which the clinical behavior weight is expressed by an individual patient^18^. Imaging loadings and behavioral loadings were obtained by Pearson’s correlation and expressed the contribution of original voxels and behavioral features to the LVs, respectively.

Bootstrap resampling was used to test the significance of imaging loadings and behavioral loadings with the replacement of 1,000 times. A bootstrap ratio for each voxel was equal to the ratio of the singular vector weight and bootstrap-estimated standard error. Thus, it can be utilized to represent the contribution of voxels to the image pattern^18^. The bootstrap ratio greater than 3.3 of brain voxels was considered statistically significant (corresponding approximately to p <0.001). The behavioral features were considered significant if the 95% confidence interval (CI) for its correlation coefficient did not cross zero.

**Reference**

1. Postuma RB, Berg D, Stern M, et al. MDS clinical diagnostic criteria for Parkinson's disease. Mov Disord. Oct 2015;30(12):1591-601. doi:10.1002/mds.26424

2. Nasreddine ZS, Phillips NA, Bedirian V, et al. The Montreal Cognitive Assessment, MoCA: a brief screening tool for mild cognitive impairment. J Am Geriatr Soc. Apr 2005;53(4):695-9. doi:10.1111/j.1532-5415.2005.53221.x

3. Weintraub D, Oehlberg KA, Katz IR, Stern MB. Test characteristics of the 15-item geriatric depression scale and Hamilton depression rating scale in Parkinson disease. Am J Geriatr Psychiatry. Feb 2006;14(2):169-75. doi:10.1097/01.JGP.0000192488.66049.4b

4. Bieling PJ, Antony MM, Swinson RP. The State-Trait Anxiety Inventory, Trait version: structure and content re-examined. Behav Res Ther. Jul-Aug 1998;36(7-8):777-88. doi:10.1016/s0005-7967(98)00023-0

5. Weintraub D, Hoops S, Shea JA, et al. Validation of the questionnaire for impulsive-compulsive disorders in Parkinson's disease. Mov Disord. Jul 30 2009;24(10):1461-7. doi:10.1002/mds.22571

6. Stiasny-Kolster K, Mayer G, Schafer S, Moller JC, Heinzel-Gutenbrunner M, Oertel WH. The REM sleep behavior disorder screening questionnaire--a new diagnostic instrument. Mov Disord. Dec 2007;22(16):2386-93. doi:10.1002/mds.21740

7. Johns MW. A new method for measuring daytime sleepiness: the Epworth sleepiness scale. Sleep. Dec 1991;14(6):540-5. doi:10.1093/sleep/14.6.540

8. Doty RL, Shaman P, Kimmelman CP, Dann MS. University of Pennsylvania Smell Identification Test: a rapid quantitative olfactory function test for the clinic. Laryngoscope. Feb 1984;94(2 Pt 1):176-8. doi:10.1288/00005537-198402000-00004

9. Goetz CG, Tilley BC, Shaftman SR, et al. Movement Disorder Society-sponsored revision of the Unified Parkinson's Disease Rating Scale (MDS-UPDRS): scale presentation and clinimetric testing results. Mov Disord. Nov 15 2008;23(15):2129-70. doi:10.1002/mds.22340

10. Hoehn MM, Yahr MD. Parkinsonism: onset, progression, and mortality. 1967. Neurology. Nov 2001;57(10 Suppl 3):S11-26.

11. Tomlinson CL, Stowe R, Patel S, Rick C, Gray R, Clarke CE. Systematic review of levodopa dose equivalency reporting in Parkinson's disease. Mov Disord. Nov 15 2010;25(15):2649-53. doi:10.1002/mds.23429

12. Yan CG, Wang XD, Zuo XN, Zang YF. DPABI: Data Processing & Analysis for (Resting-State) Brain Imaging. Neuroinformatics. Jul 2016;14(3):339-51. doi:10.1007/s12021-016-9299-4

13. Power JD, Barnes KA, Snyder AZ, Schlaggar BL, Petersen SE. Spurious but systematic correlations in functional connectivity MRI networks arise from subject motion. Neuroimage. Feb 1 2012;59(3):2142-54. doi:10.1016/j.neuroimage.2011.10.018

14. Cartmell SC, Tian Q, Thio BJ, et al. Multimodal characterization of the human nucleus accumbens. Neuroimage. Sep 2019;198:137-149. doi:10.1016/j.neuroimage.2019.05.019

15. Yu M, Linn KA, Cook PA, et al. Statistical harmonization corrects site effects in functional connectivity measurements from multi-site fMRI data. Hum Brain Mapp. Nov 2018;39(11):4213-4227. doi:10.1002/hbm.24241

16. Li K, Fan L, Cui Y, et al. The human mediodorsal thalamus: Organization, connectivity, and function. Neuroimage. Apr 1 2022;249:118876. doi:10.1016/j.neuroimage.2022.118876

17. Rahayel S, Postuma RB, Montplaisir J, et al. A Prodromal Brain-Clinical Pattern of Cognition in Synucleinopathies. Ann Neurol. Feb 2021;89(2):341-357. doi:10.1002/ana.25962

18. Zeighami Y, Fereshtehnejad SM, Dadar M, et al. A clinical-anatomical signature of Parkinson's disease identified with partial least squares and magnetic resonance imaging. Neuroimage. Apr 15 2019;190:69-78. doi:10.1016/j.neuroimage.2017.12.050
